# Diagnostic and prognostic value of plasma miR-106a-5p levels in patients with acute heart failure

**DOI:** 10.1186/s13019-024-02750-7

**Published:** 2024-04-23

**Authors:** Aike Fei, Li Li, Yunfang Li, Tie Zhou, Yanfei Liu

**Affiliations:** 1 Department of Cardiovascular Medicine, The Fourth Hospital of Changsha, Changsha Hospital of Hunan Normal University, No. 70, Lushan Road, Yuelu District, Changsha, Hunan Province 410006 China; 2 Cardiovascular Specialist, Community Health Service Center, No. 668, Minghutang Group, Hanpu Street, Yuelu District, Changsha City, Hunan Province 410006 China

**Keywords:** Acute heart failure, miR-106a-5p, Poor prognosis, ROC curve, NT-proBNP, hs-CRP

## Abstract

**Background:**

It is essential to find reliable biomarkers for early diagnosis and prognosis of acute heart failure (AHF) for its mitigation. Currently, increasing attention is paid to the role of microRNAs (miRNAs/miRs) as diagnostic or prognostic markers for cardiovascular diseases. Since plasma miR-106a-5p has been observed to be downregulated in AHF, its value in the diagnosis and prognostic assessment of AHF deserves further exploration. Accordingly, this study analyzed the diagnostic and prognostic value of plasma miR-106a-5p in AHF patients.

**Methods:**

Prospectively, this study included 127 AHF patients who met the 2021 European Society of Cardiology Guidelines and 127 control individuals. Plasma miR-106a-5p levels were determined with RT-qPCR. Spearman correlation analysis was performed to evaluate the correlation of plasma miR-106a-5p levels with NT-proBNP and hs-CRP levels in AHF patients. All AHF patients were followed up for 1 year and allocated into poor and good prognosis groups, and plasma miR-106a-5p levels were compared. The diagnostic and prognostic value of plasma miR-106a-5p for AHF was assessed with a receiver-operating characteristic curve.

**Results:**

Plasma miR-106a-5p was lowly expressed in AHF patients versus controls (0.53 ± 0.26 vs. 1.09 ± 0.46) and showed significant negative correlations with NT-proBNP and hs-CRP levels. Plasma miR-106a-5p level < 0.655 could assist in AHF diagnosis. Plasma miR-106a-5p levels were markedly lower in poor-prognosis AHF patients than in good-prognosis patients. Plasma miR-106a-5p level < 0.544 could assist in predicting poor prognosis in AHF patients.

**Conclusion:**

Plasma miR-106a-5p is downregulated in AHF patients and could assist in diagnosis and poor prognosis prediction of AHF.

**Supplementary Information:**

The online version contains supplementary material available at 10.1186/s13019-024-02750-7.

## Introduction

Heart failure (HF) is a chronic disease caused by defects in myocardial function that can lead to impaired ventricular filling or ejection [1]. It is a primary contributor to poor quality of life, mortality, and morbidity [2]. Acute HF (AHF) refers to new or worsening symptoms and signs of HF and is the most common cause of unplanned hospital admission in patients aged over 65 years [3], which primarily manifests as signs and symptoms associated with systemic congestion, namely extracellular fluid accumulation due to elevated biventricular cardiac filling pressures [4]. Early diagnosis and intervention for individuals at high risk of suspected AHF may alleviate their conditions [5]. However, the diagnosis of AHF solely with clinical presentations is a challenge, and diagnostic biomarkers are needed.

Currently, B-type natriuretic peptide (BNP) and N-terminal proBNP (NT-proBNP) are extensively utilized as diagnostic biomarkers for AHF in clinical practice [6, 7]. Because of its longer half-life and higher stability, NT-proBNP is more suitable for clinical application than BNP. Additionally, the high level of NT-proBNP can, to a certain extent, predict the prognosis of HF patients. However, given that its level is readily affected by some non-cardiac diseases, the application of NT-proBNP alone is ineffective, and other indicators are often used as a supplement in clinical practice [8]. Therefore, it is critical to investigate other biomarkers as complementary tools to NT-proBNP and hs-CRP for the early diagnosis and prognostic evaluation of AHF.

microRNAs (miRNAs/miRs) are highly conserved non-coding RNAs involved in many cellular functions and physiological processes [9, 10]. There is growing evidence for the role of miRNAs as diagnostic or prognostic markers in various clinical settings [11]. Aberrant expression of miRNAs is closely associated with AHF, which may provide new strategies for early diagnosis, severity grading, and prognosis of AHF [12–14]. For example, circulating miR-19b-3p may be a new prognostic parameter for AHF, and high levels of circulating miR-19b-3p may imply ventricular hypertrophy in AHF patients [15]. In addition, plasma miR-212-3p can be used as a biomarker for acute right HF with pulmonary hypertension [16]. Serum miR-30d levels are markedly reduced in AHF patients and can be used to predict survival in AHF patients [17]. miR-106a-5p is closely associated with diverse cardiovascular diseases such as myocardial ischemia/reperfusion injury and coronary artery disease [18, 19]. It is also a promising marker for acute myocardial infarction [20, 21]. Recent research demonstrated the downregulation of plasma miR-106a-5p in AHF patients [12]. However, it is uncertain about the value of plasma miR-106a-5p levels for early diagnosis and prognostic assessment of AHF.

Therefore, this study investigated plasma miR-106a-5p expression and its diagnostic and prognostic value in AHF patients, thus providing references for further clarification of AHF pathogenesis and new ideas for clinical prediction and management of AHF.

## Methods

### Participants

In this study, we prospectively selected 183 consecutive AHF patients admitted to the Fourth Hospital of Changsha from January 2020 to April 2022 for screening (all patients were screened only once on admission). Among these patients, there were 39 patients not meeting the inclusion criteria, 12 patients reluctant to participate in the study, and 5 patients with incomplete information. Finally, 127 AHF patients were included in this study. The control group included 127 individuals who underwent preventive physical examinations in our hospital during the same period and were matched with AHF patients in terms of gender, body mass index (BMI), systolic blood pressure (SBP), and diastolic blood pressure (DBP). This study was conducted following the *Declaration of Helsinki* and approved by the Academic Ethics Committee of the Fourth Hospital of Changsha. The patients were informed and signed a consent form. (Approval number: CSSDSYY-YXLL-SC-2024-01-94)

### Inclusion and exclusion criteria

Inclusion criteria for AHF patients were as follows: (1) patients meeting the diagnostic criteria of AHF in the European Society of Cardiology (ESC) guidelines [22]; (2) patients with New York Heart Association Cardiac Function classes of II-IV [15]; (3) patients with complete clinical data; (4) patients aged > 18 years; (5) patients with complete follow-up data; (6) patients who gave informed consent for the study and patients aged 60 years or above whose families also provided informed consent.

Inclusion criteria for control individuals were as follows: (1) individuals with complete clinical data; (2) individuals aged > 18 years; (3) individuals matched with AHF patients in terms of gender, BMI, SBP, and DBP; and (4) individuals who gave informed consent for this study.

Exclusion criteria for AHF patients or control individuals were as follows: (1) patients or individuals with acute myocardial infarction or severe arrhythmia combined with severe valvular stenosis; (2) patients or individuals with severe immune or hematological diseases; (3) patients or individuals with malignant tumor; (4) patients or individuals with severe internal diseases or infections; (5) patients or individuals who suffered from mental disorders and failed to cooperate with follow-up.

### Data collection

The following clinical information of all participants at enrolment was recorded: age, gender, BMI, alcohol consumption history (alcohol consumption for more than five years, with daily alcohol intake of ≥ 40 g for men and ≥ 20 g for women, or heavy alcohol consumption in the last two weeks, with daily alcohol intake of ≥ 80 g), smoking history, diabetes mellitus, hypertension, SBP, and DBP. The left atrial structure and function were assessed using a German Philips IE33 ultrasound imager with an S4 ultrasound probe at 2.5 MHz. Participants were placed in the left lateral position. After fixation of two-dimensional left ventricular (LV) long-axis images, the LV end-diastolic internal diameter (LVEDD) and left atrial diameter (LAD) were measured, and LV ejection fraction (LVEF) was calculated: LVEF = (end-diastolic volume [EDV]-end-systolic volume [ES]) × 100%/EDV.

### Enzyme-linked immunosorbent assay (ELISA)

The levels of high-sensitivity C-reactive protein (hs-CRP; EY-01H1141) and NT-proBNP (FT-P32955R) in the plasma of all participants were detected with ELISA as instructed in the manuals of hs-CRP (Shanghai Yiyan Biotechnology Co., Ltd., Shanghai, China) and NT-proBNP (Shanghai Fantai Biotechnology Co., Ltd., Shanghai, China) kits.

### Reverse transcription-quantitative polymerase chain reaction (RT-qPCR)

Briefly, 5 mL of peripheral venous blood was drawn from each participant early in the morning of the next day of enrollment, placed in ethylene diamine tetraacetic acid anticoagulation tubes, and centrifuged at 1000 × g for 10 min at 4 °C to obtain plasma. All samples were preserved in RNase-free microcentrifuge tubes and stored in a -80 °C refrigerator, and RT-qPCR was performed within 1 week. Specifically, total RNA was extracted from plasma with TRIzol reagents (Thermo Fisher Scientific, Waltham, MA, USA) and isolated with miRNeasy Serum/Plasma Kit (QIAGEN, Hilden, Germany; 217,184) [23], followed by the measurement of RNA purity and concentration on a NanoDrop 2000 spectrophotometer (Thermo Fisher Scientific). cDNA was synthesized from 500 ng RNA with an RT System Kit (Takara, Dalian, China). Next, RT-qPCR was carried out with SYBR Green PCR Mix (Takara): 94 °C for 30 s, 94 °C for 10 s, and 60 °C for 30 s for 40 cycles. The relative level of miR-106a-5p was normalized by that of U6 and calculated with the 2^−ΔΔCt^ method. The primers are listed in Table 1.

**Table 1:** RT-qPCR primer sequences

Gene	Forward 5’-3’	Reverse 5’-3’
miR-106a-5p	TCCAGCTGGGCCCAGTGTTCAGACTAC	GTGTCGTGGAGTCGGCAATTC
U6	GCTTCGGCAGCACATATACTAAAAT	CGCTTCACGAATTTGCGTGTCAT

### Follow-up

All AHF patients were given standardized treatment with reference to the ESC Guidelines [22]. Patients were followed up every 3 months after discharge by telephone or in an outpatient clinic for 1 year to record the prognosis of patients. Failure to respond to the phone was recorded as loss to follow-up. The poor prognosis was defined as cardiovascular disease-related death (CV-related death) or readmission due to exacerbation of HF [24]. AHF patients were classified into good prognosis (*n* = 91) and poor prognosis (*n* = 36) groups.

### Statistical analysis

Data were statistically analyzed and graphed with SPSS 21.0 and GraphPad Prism 6.0 software. The Shapiro-Wilk test was utilized to test the normal distribution of data. Data that conformed to normal distribution were depicted as mean ± standard deviation, and pairwise comparisons were conducted with the independent samples *t*-test. Data without normal distribution were summarized as median (minimum, maximum) and analyzed with the Mann-Whitney test. Categorical variables were analyzed with the Fisher’s exact test. The correlation between miR-106a-5p and the levels of NT-proBNP and hs-CRP was assessed with the Spearman correlation analysis. The diagnostic and prognostic value of miR-106a-5p for AHF was analyzed with the receiver-operating characteristic (ROC) curve. *P* < 0.05 indicated a statistically significant difference.

## Results

### Comparative analysis of baseline clinical data

There was no statistical difference between the AHF and control groups in terms of age, gender, BMI, alcohol consumption history, smoking history, diabetes mellitus, SBP, and DBP (all *P* > 0.05). Statistically significant differences were noted in hypertension, LAD, LVEDD, LVEF, NT-proBNP, and hs-CRP between the two groups (all *P* < 0.05) (Table 2).

**Table 2:** Comparative Analysis of Clinical Baseline Data

Characteristics	Control (*n* = 127)	AHF (*n* = 127)	*P* value
Age	61 (46,73)	62 (47,76)	0.535
Gender [Male, n (%)]	69 (54.33%)	74 (58.27%)	0.613
BMI (kg/m^2^)	25.57 ± 3.12	25.65 ± 2.97	0.834
Smoking [n (%)]	49 (38.58%)	54 (42.52%)	0.609
Alcohol consumption [n (%)]	60 (47.24%)	56 (44.09%)	0.706
Type 1 and 2 diabetes mellitus [n (%)]			
Type 1	4 (19.05%)	4 (12.50%)	0.515
Type 2	17 (80.95%)	28 (87.50%)	
Hypertension [n (%)]	58 (45.67%)	79 (62.20%)	0.012
SBP (mmHg)	121.08 ± 10.27	123.24 ± 12.86	0.140
DBP (mmHg)	75.14 ± 7.52	76.31 ± 7.65	0.220
LAD (mm)	36.42 ± 3.96	40.16 ± 4.87	< 0.001
LVEDD (mm)	47.73 ± 6.04	49.72 ± 4.61	0.004
LVEF (%)	61.18 ± 7.56	32.62 ± 5.74	< 0.001
NT-proBNP (pg/mL)	25.75 (16.60, 35.36)	102.97 (67.63, 156.34)	< 0.001
hs-CRP (mg/L)	5.18 (3.01, 7.12)	8.29 (4.30, 13.36)	< 0.001

*Note* AHF: acute heart failure; BMI: body mass index; SBP: systolic blood pressure; DBP: diastolic blood pressure; LAD: left atrium dimension; LVEDD: left ventricular end-diastolic diameter; LVEF: left ventricular ejection fraction; NT-proBNP: N-terminal pro-B type natriuretic peptide; hs-CRP: hypersensitive C-reactive protein. The Shapiro-Wilk test was used for normal distribution. Measurements that conformed to normal distribution were expressed as mean ± standard deviation and analyzed by independent samples t-test; data that did not conform to normal distribution were expressed as median (minimum, maximum) by Mann-Whitney test; categorical variables were expressed as n (%) by Fisher’s exact test. *P* < 0.05 indicated that the difference was statistically significant

### Low plasma miR-106a-5p levels in AHF patients

**Fig. 1:**
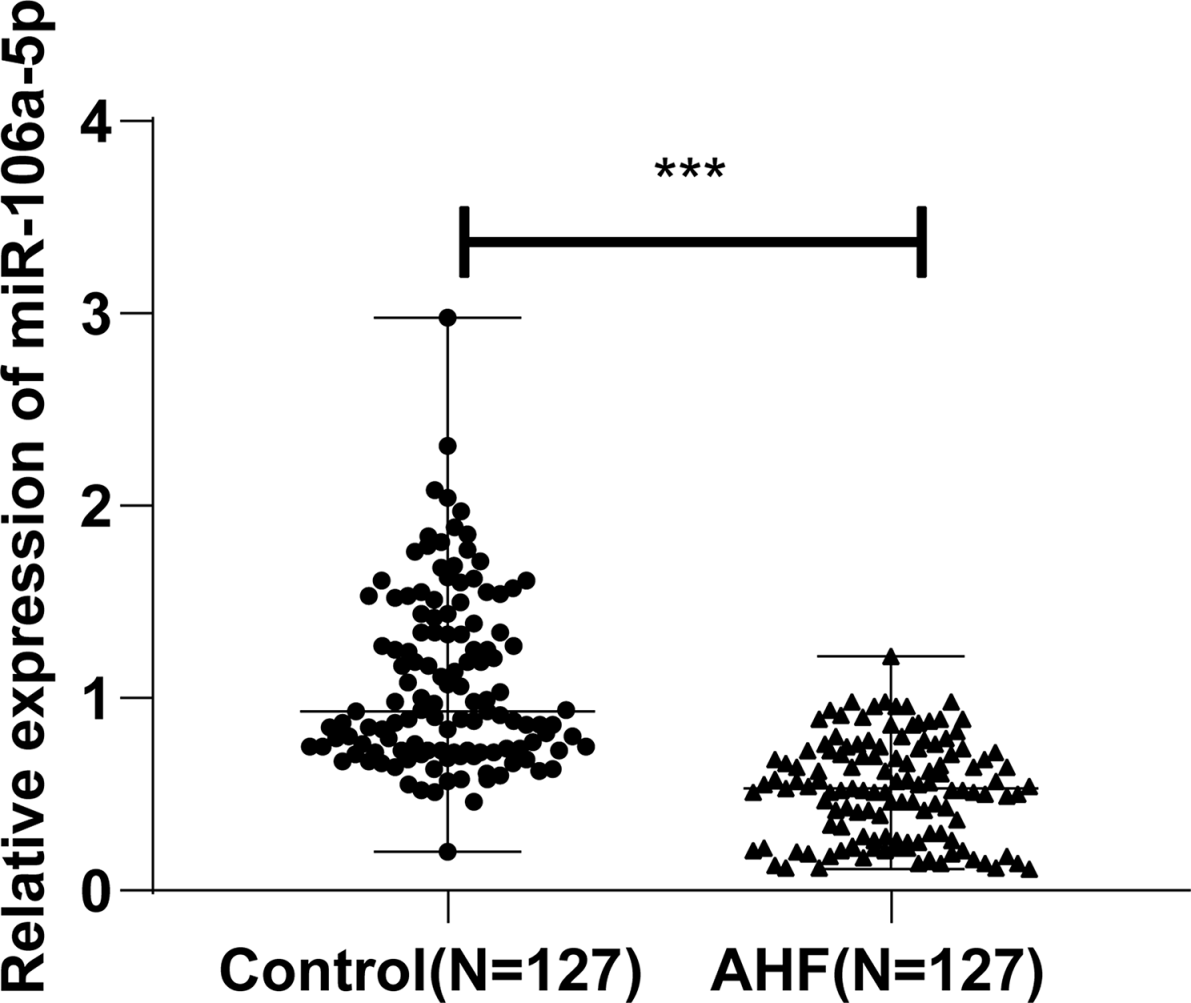
Low expression of plasma miR-106a-5p in AHF patients. *Note* miR-106a-5p expression was detected with RT-qPCR. Measurement data were expressed as median (minimum, maximum), and data between the two groups were compared with the Mann-Whitney test. *** *P* < 0.001

RT-qPCR showed lower plasma miR-106a-5p levels in the AHF group than in the control group (*P* < 0.001, Fig. 1 and Table S1), illustrating that miR-106a-5p is lowly expressed in the plasma of AHF patients.

### Correlation analysis of plasma miR-106a-5p levels with NT-proBNP and hs-CRP levels in AHF patients

**Fig. 2:**
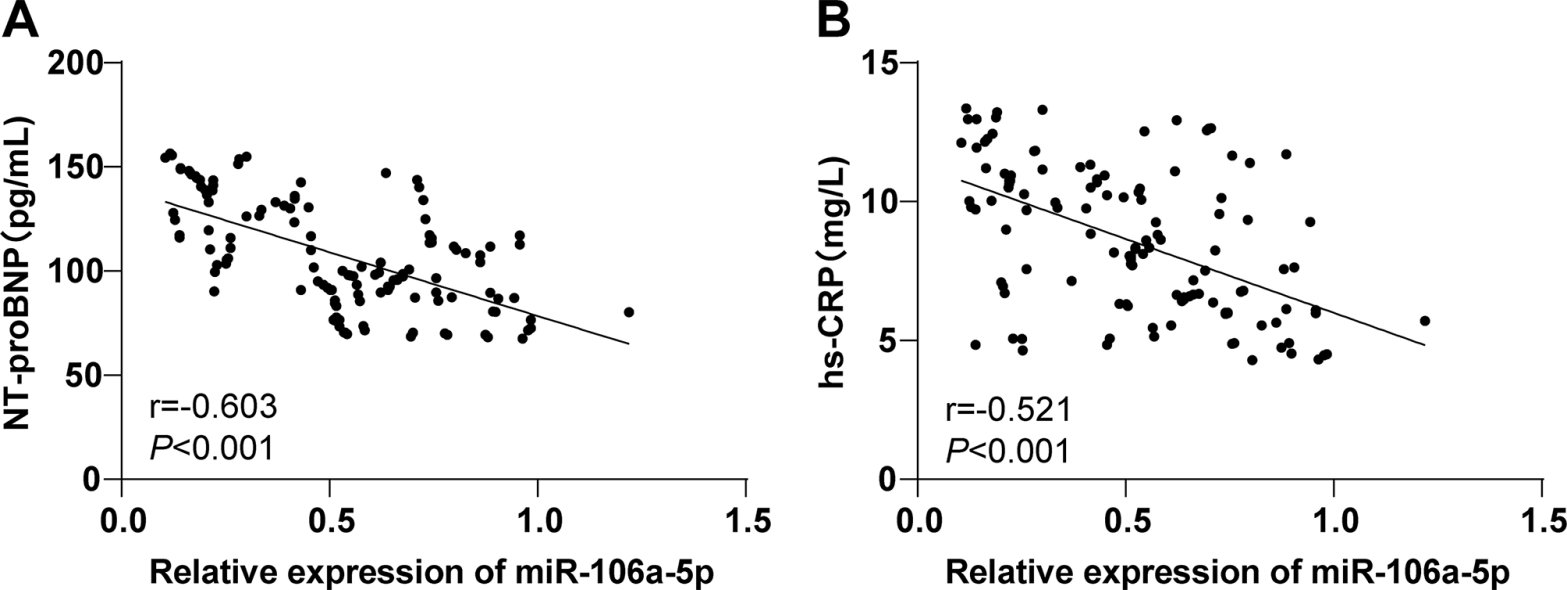
Correlation analysis of plasma miR-106a-5p levels with NT-proBNP and hs-CRP levels in AHF patients. *Note* Spearman correlation analysis of plasma miR-106a-5p levels with plasma NT-proBNP (A) and hs-CRP (B) levels in AHF patients. r is the correlation coefficient, and *P* < 0.05 indicates a statistically significant difference

As found in previous research, NT-proBNP and hs-CRP are highly expressed in the serum of HF patients [25, 26] and are of great reference value in assisting the diagnosis of AHF [6, 27]. Therefore, the Spearman correlation analysis was performed to explore the correlation of plasma miR-106a-5p levels with NT-proBNP and hs-CRP levels in AHF patients. Plasma miR-106a-5p levels were negatively linked to plasma levels of NT-proBNP (*r* = -0.603, *P* < 0.001, Fig. 2A) and hs-CRP (*r* = -0.521, *P* < 0.001, Fig. 2B) in AHF patients.

### Low plasma miR-106a-5p expression aids in the diagnosis of AHF

**Fig. 3:**
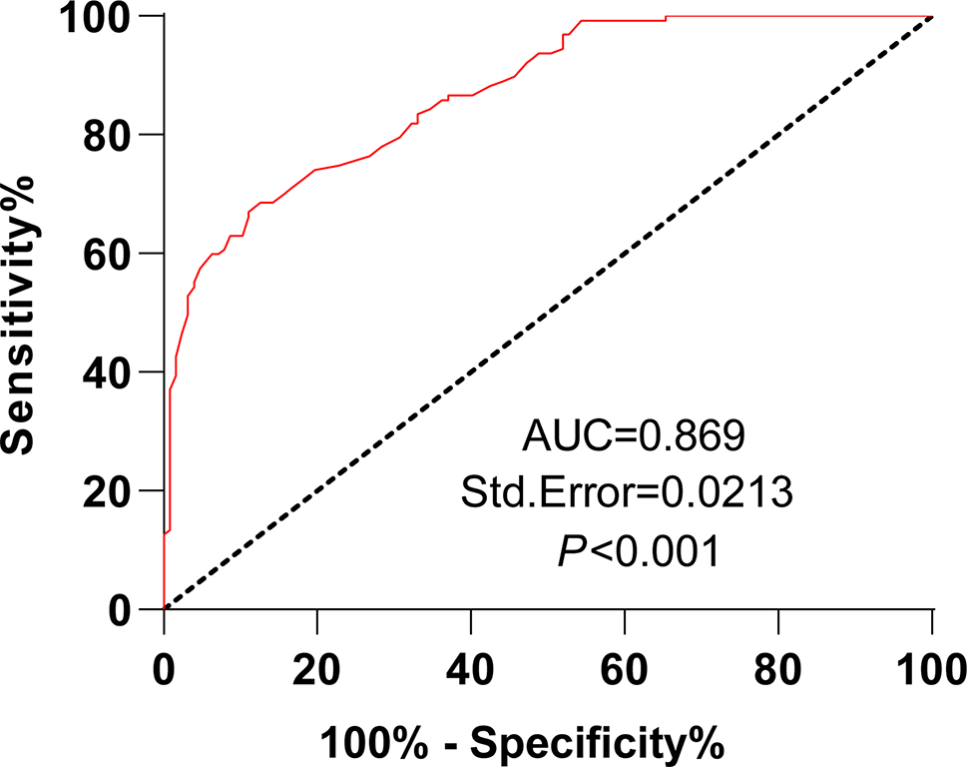
Low plasma miR-106a-5p expression aids in the diagnosis of AHF. *Note* ROC curve analysis was performed to assess the diagnostic vale of plasma miR-106a-5p levels for the development of AHF

This study further assessed the diagnostic value of plasma miR-106a-5p levels for AHF. The ROC curve exhibited that the area under the ROC curve (AUC) of plasma miR-106a-5p levels for the diagnosis of AHF was 0.869, with a cut-off value of 0.655, a sensitivity of 66.93%, and a specificity of 88.98%, indicating that plasma miR-106a-5p level < 0.655 could assist in AHF diagnosis (Fig. 3).

### Low plasma miR-106a-5p levels in poor-prognosis AHF patients

**Fig. 4:**
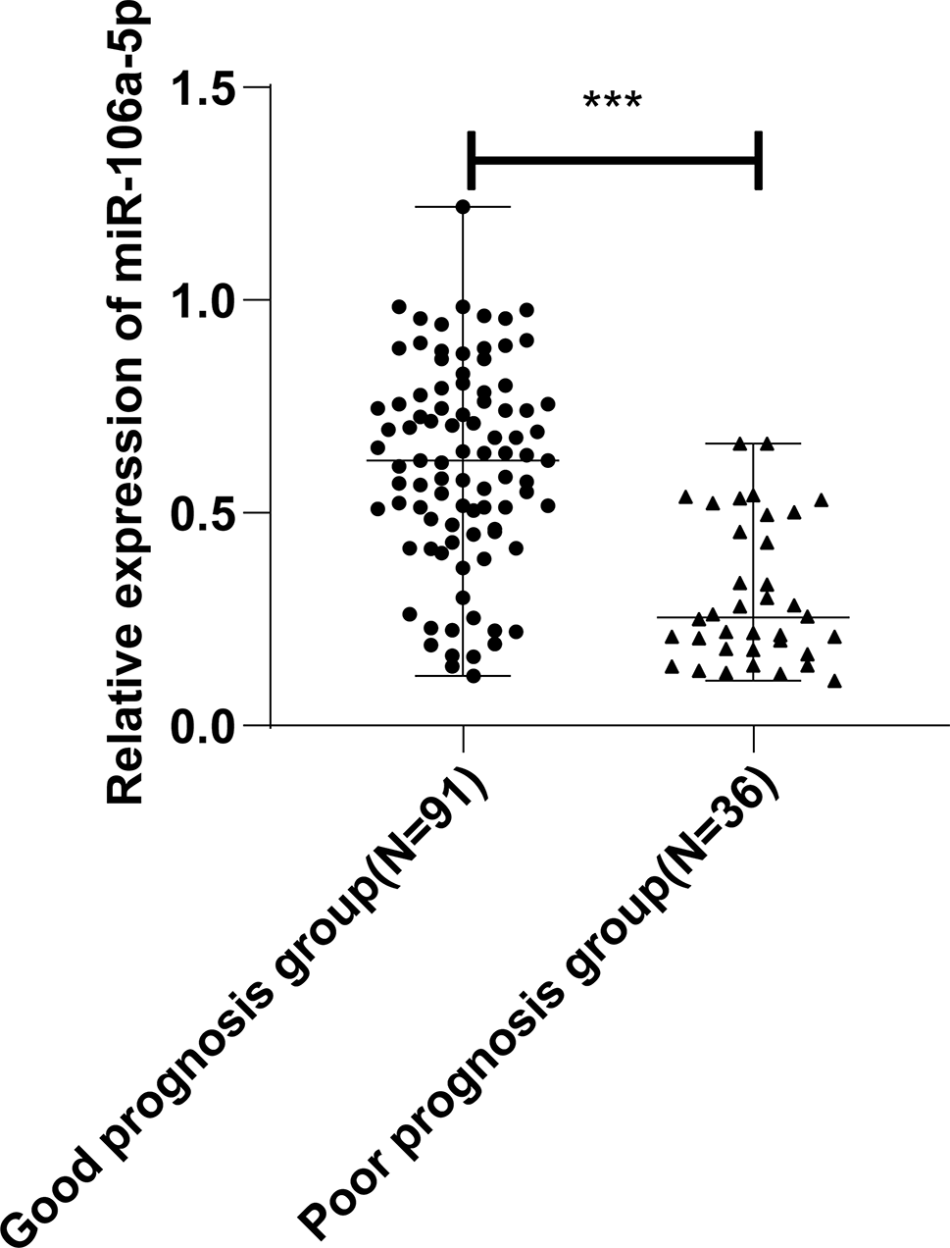
Differences in plasma miR-106a-5p levels between good and poor prognosis groups of AHF patients. *Note* miR-106a-5p expression was detected with RT-qPCR. Measurement data were expressed as median values (minimum, maximum), and data were compared between the two groups with the Mann-Whitney test. *** *P* < 0.001

AHF patients were followed up every 3 months after discharge for 1 year to record the prognosis and then categorized into good prognosis (*n* = 91) and poor prognosis (*n* = 36) groups. In the poor prognosis group (*n* = 36), there were 21 (58.33%) patients with readmission for AHF and 15 (41.67%) patients with CV-related death. RT-qPCR demonstrated that plasma miR-106a-5p levels were markedly lower in the poor prognosis group than in the good prognosis group (*P* < 0.001, Fig. 4).

### Low plasma miR-106a-5p expression aids in predicting poor prognosis in AHF patients

**Fig. 5:**
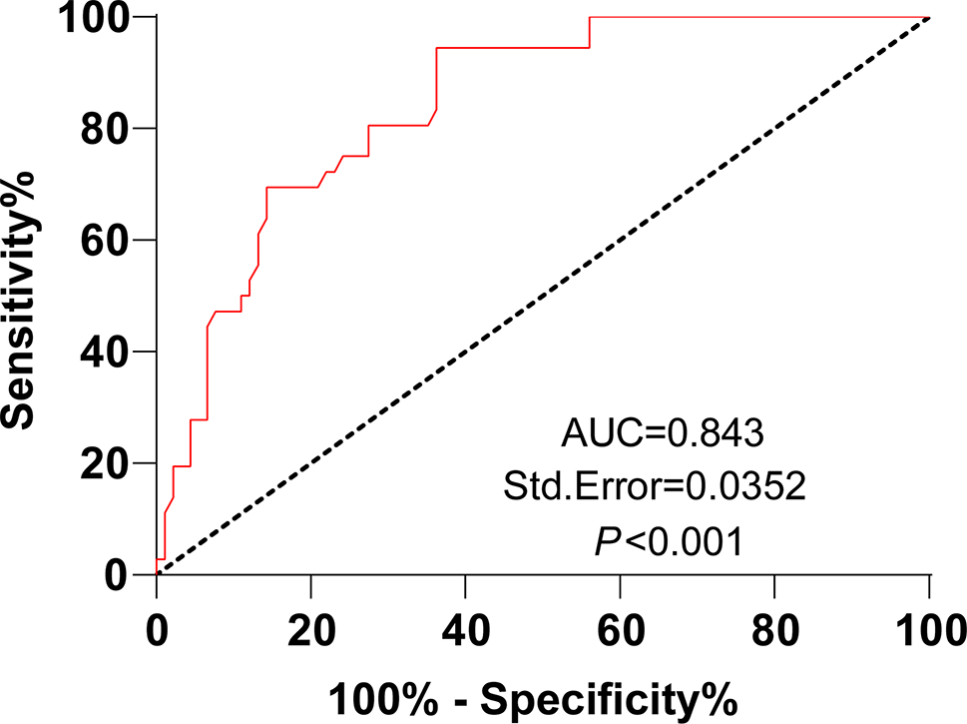
Low plasma miR-106a-5p expression aids in predicting poor prognosis in AHF patients. *Note* ROC curve was used to assess the value of plasma miR-106a-5p levels in predicting poor prognosis in AHF patients

Subsequently, this study further analyzed the predictive value of plasma miR-106a-5p levels for the prognosis of AHF patients. The ROC curve revealed that the AUC of plasma miR-106a-5p levels in predicting poor prognosis was 0.843, with a cut-off value of 0.544, a sensitivity of 94.44%, and a specificity of 63.74%, which highlighted that plasma miR-106a-5p level < 0.544 could assist in predicting poor prognosis in AHF patients (Fig. 5).

## Discussion

AHF is regarded as new or worsening HF symptoms and signs and is related to high rates of mortality and hospital readmission, generally accompanied by poor outcomes [4]. In comparison to chronic HF, there is limited evidence to guide the accurate diagnosis and management of AHF [28]. This harsh reality forces researchers to conduct more investigations on AHF, especially in terms of diagnosis and prognosis. A great body of available literature has demonstrated the significance of miRNAs for the diagnosis and prognosis of heart diseases, including HF and myocardial infarction [29–34]. The salient findings from this study illustrated that plasma miR-106a-5p expression was reduced in AHF patients and could assist in diagnosing AHF and predicting the poor prognosis of AHF.

It is noteworthy that miRNAs confer imperative functions in cardiovascular diseases (including HF) and are implicated in an array of pathophysiological processes, such as hypertrophy and cardiac fibrosis, with potential as diagnostic and prognostic markers for HF [35]. Accumulating reports have stated the dysregulated expression patterns of miRNAs in HF individuals [36, 37]. Plasma miR-106a-5p is notably downregulated in AHF patients relative to healthy controls [12]. Consistently, RT-qPCR data in our study confirmed the down-regulation of plasma miR-106a-5p in AHF patents, implicating that low expression of miR-106a-5p is involved in AHF development. As reported, miR-106a-5p is an AHF-specific miRNA and is related to the predicted targets and pathways associated with cardiac disorders, such as cardiac fibrosis/remodeling, angiogenesis, and inflammation [34]. While the development of AHF is associated with hemodynamic abnormalities and inflammatory activation [38, 39]. Moreover, NT-proBNP is established as a blood biomarker for the diagnosis and prognosis of HF, and CRP is identified as a common inflammatory factor in HF, both of which are elevated in patients with HF and valvular heart disease [40–43]. Accordingly, it could be speculated that miR-106a-5p expression might be negatively correlated with NT-proBNP and CRP levels in HF. Furthermore, our findings revealed that plasma miR-106a-5p levels exhibited a negative correlation with NT-proBNP and hs-CRP levels. Based on these studies and results, miR-106a-5p downregulation may facilitate hemodynamic abnormalities and inflammation to drive AHF progression.

Compelling evidence reveals that circulating miRNAs, consisting of miR-106a-5p, hold the potential to predict future fatal myocardial infarction in healthy individuals [21]. miR-106a-5p displays a high AUC value for AHF and is negatively linked to creatinine, a biomarker reflecting poor clinical outcomes of worsening AHF patients at 48 h of hospitalization [34]. Overall survival is short in HF patients with reduced serum levels of miR-106a-5p [8]. A further reduction in miR-106a-5p expression during the first 48 h of hospitalization is linked to an elevated risk of 180-day mortality [12]. Low miR-106a-5p level is related to worsening renal function in AHF patients [44]. These studies highlight that miR-106a-5p reflects the poor prognosis of AHF and may be used to predict the prognosis of patients. Considering these studies, our research further delved into the clinical performance of plasma miR-106a-5p in the diagnosis and prognostic prediction of AHF. Our results displayed that plasma miR-106a-5p levels could aid the diagnosis of AHF (AUC: 0.869, cut-off value: 0.655, sensitivity: 66.93%, and specificity: 88.98%) and the prediction of unfavorable outcomes in AHF patients (AUC: 0.843, cut-off value: 0.544, sensitivity: 94.44%, and specificity: 63.74%). A prior study showed that the AUC of miR-106a-5p for the diagnosis of AHF was in the range of f 0.82–0.97 [34], similar to the AUC in our study. Overall, miR-106a-5p has a higher diagnostic value for AHF patients. Our results also elucidated that AHF patients with poor prognoses had lower plasma miR-106a-5p levels than those with good prognoses. Different from the aforementioned studies, our study exhibited the specific performance of plasma miR-106a-5p in the diagnosis and prognostic prediction of AHF by analyzing AUC, sensitivity, and specificity and the negative correlation of plasma miR-106a-5p levels with NT-proBNP and hs-CRP levels in AHF patients. A previous study unraveled that the miR-106a-363 cluster fostered endogenous myocardial repair in heart injury by targeting Notch3 [45]. Notch activation has been reported to be associated with the poor prognosis of HF [46]. Hence, the target genes of miR-106a-5p deserve exploration to further clarify the pathogenesis of AHF.

In conclusion, this prospective study underlined that plasma miR-106a-5p levels were decreased in AHF patients and negatively related to NT-proBNP and hs-CRP levels. Moreover, ROC curve analysis and 1-year follow-up for AHF patients unveiled that low expression of plasma miR-106a-5p could assist in diagnosing AHF and predicting the poor prognosis of AHF. The above findings offer novel insight into clinical diagnosis, condition judgment, and prognosis prediction of AHF. Nevertheless, there are several limitations to this study. First, the number of included cases and events is relatively small. Second, the basic regulatory mechanisms of miR-106a-5p in AHF development were not thoroughly investigated. In the future, multi-center prospective research with more samples is needed to increase the credibility of these results. Third, peripheral venous blood was drawn from all patients in the morning of the day following enrollment, which implies that miRNA concentrations may be affected by the therapies used for AHF patients. Therefore, further studies involving the controlling of blood collection time are warranted to eliminate the possible effect of therapy on miR-106a levels. Fourth, it is worthy to analyze the correlation between plasma miR-106a-5p levels and clinical criteria such as disease stage in AHF patients.

### Electronic supplementary material

Below is the link to the electronic supplementary material.

Supplementary Material 1

## Data Availability

No datasets were generated or analysed during the current study.
